# Influence of Comorbidities on Colorectal Cancer Screening Participation and Mortality

**DOI:** 10.1016/j.ajmo.2025.100123

**Published:** 2025-12-14

**Authors:** Rachel Corren, Sylvia La, Edgar Corona, Dalia Martinez, Urmimala Sarkar, Blake Gregory, Uri Ladabaum, Ma Somsouk

**Affiliations:** a School of Medicine, University of California, San Francisco, Calif; b San Francisco Health Network, San Francisco Department of Public Health, San Francisco, Calif; c Division of Gastroenterology, University of California, San Francisco, Calif; d Clinical Translational Science Institute, University of California, San Francisco, Calif; e Division of General Internal Medicine, University of California, San Francisco, Calif; f Division of Gastroenterology and Hepatology, Stanford University School of Medicine, Stanford, Calif; g Action Research Center for Health Equity, University of California, San Francisco, Calif

**Keywords:** Colorectal cancer, Fecal immunochemical test, Organized screening, Population health

## Abstract

**Aims:**

Organized screening programs improve colorectal cancer (CRC) screening participation, but outreach services can be improved. We sought to understand screening deferral by examining patient-level factors and how they relate to fecal immunochemical test (FIT) orders, completion rates, and long-term mortality.

**Methods:**

Patients aged 50-75 years who were not up to date with CRC screening receiving usual care were followed over time (NCT02613260). Patient-level laboratory and cancer registry data were used to identify patients who met a specified laboratory threshold: albumin < 3 g/dL, HIV viral load > 10,000 copies or CD4 < 200 cells/µL, creatinine > 4 mg/dL, platelets < 100,000/µL, total bilirubin > 4 µmol/L, NH3 > 20, positive urine amphetamine or cocaine, serum ethanol > 80, hemoglobin A1C > 10%, and stage 3 or 4 cancer. The proportion of patients with a FIT order, FIT completion in 1-year, and mortality at 8-years were compared in patents with and without the lab abnormality.

**Results:**

Nine thousand six hundred seventy-six patients were eligible for screening, of which 1053 met the criteria for laboratory abnormalities. Patients with laboratory abnormalities were less likely to have a FIT order placed (39.5% vs 66.8%, *P* < .001) and were less likely to complete FIT screening (21.5% vs 51.6%, *P* < .001). Moreover, patients with laboratory abnormalities experienced higher mortality at 8-year follow-up (32.6% vs 6.7%, *P* < .001).

**Conclusions:**

Patients with laboratory abnormalities were less likely to have a FIT order placed and completed, and experienced higher mortality, suggesting that screening was deferred by providers. Future studies should gather provider input to understand how patient-level electronic data could be considered in the implementation of screening services.

## Highlights

• Centrally organized screening outreach programs increase colorectal cancer (CRC) screening participation, but outreach services can be improved.
• An agnostic screening invitation, in which every patient is invited to screen, may not be consistent with the intent of primary care providers.
• This study showed that patients meeting abnormal lab thresholds were much less likely to receive an order for colorectal cancer screening, less likely to complete screening, and experienced higher mortality.
• Health systems can enhance the delivery of an organized screening program by incorporating patient-level factors to guide outreach services.
• Greater trust in organized screening programs would increase adoption, which could in turn improve overall screening rates.

## Introduction

Colorectal cancer (CRC) screening, currently recommended for ages 45-75, leads to early detection and prevention of colon cancer.^1^ The fecal immunochemical test (FIT) is a recommended screening modality that has been increasingly adopted by integrated health systems due to its performance characteristics,^2^ programmatic effectiveness,^3^ and patient acceptability.^4^ Health systems have increasingly leveraged digital health records to implement FIT outreach programs.^5^

Despite its public health importance, CRC screening is not appropriate for all individuals. For example, guidelines recommend that clinicians selectively offer screening for patients with limited life expectancy.^6^, ^7^^–^^8^ Similarly, the Center for Medicare Services (CMS) Quality Payment Program’s measure for appropriate CRC screening allows for the exclusion of patients from the measure if they have a claim for frailty, acute inpatient encounter, advanced illness diagnosis, or use of dementia medications.^9^ Clinicians understand that the benefits of screening are moderated in patients with comorbidities who are likely to die from causes other than CRC. Ideally, population-based outreach programs would be aligned with patient and provider preferences for and deferral of screening. However, many organized outreach programs have not instituted formal methods to defer patients from routine screening invitations who have significant coexisting comorbidities.^10^

In this study, we conducted a secondary analysis of patients receiving usual care from a defined cohort assembled as part of an interventional randomized trial (NCT02613260). The cohort of patients received usual care and we identified a subset meeting a specified laboratory abnormality. We examined the FIT order as a proxy of primary care clinician assessment and intent, which may reflect shared decision-making with the patient. FIT completion, which follows a FIT order, further captures the patients’ own self-assessment. Higher all-cause mortality in the group with laboratory abnormalities would validate the idea that screening is less applicable in this group. Therefore, we hypothesized that patients with comorbidities derived from structured health data (ie laboratory data) were less likely to have a FIT order and complete the FIT when ordered, while experiencing excess mortality. If so, this would provide evidence in support of incorporating patient-level data in organized screening programs to target screen-eligible populations while deferring others as intended by their primary care team.

## Methods

### Health Care Setting

The current study is a secondary analysis of patients receiving usual care from a defined cohort assembled as part of an interventional randomized trial. This cohort was assembled in 2016, consisting of patients 50-75 years of age in the San Francisco Health Network (SFHN) who were identified as being potentially eligible for CRC screening. SFHN is a publicly funded integrated safety-net health system providing services to low-income populations. It consists of 12 adult primary care clinics and 1 specialty medical center, Zuckerberg San Francisco General Hospital. These clinics share an electronic health record system, 1 clinical laboratory, and refer to 1 gastroenterology unit.

### Patient Population, Predictors, Outcomes

The parent study deriving this cohort included patients aged 50-75 years who were not up to date with CRC screening (NCT02613260). Data from the trial NCT02613260 were accessed through a database available to study authors (MS, RC) and can be accessed by request from external groups. In the parent study, patients were randomly assigned to 2 groups, 1 group to receive organized screening outreach via mailed FIT vs another to usual care; this randomization occurred after deferring a subset of patients with laboratory abnormalities in the preceding year. The study team, with primary care input, utilized laboratory values and the cancer pathology registry to define patients with lab-based abnormalities that should be deferred from randomization and the mailed outreach intervention; instead, screening could still occur at the discretion of the primary care provider. Patients with significant laboratory abnormalities were defined as having at least 1 of the following: albumin < 3 g/dL, HIV viral load > 10,000 copies or CD4 < 200 cells/µL, creatinine > 4 mg/dL, platelets < 100,000/µL, total bilirubin > 4 µmol/L, NH_3_ > 20, positive urine amphetamine or cocaine, serum ethanol > 80, hemoglobin A1C > 10%, and stage 3 or 4 cancer.

The current study focuses on the patients assigned to usual care and the subset of patients with laboratory abnormalities deferred from randomization. Neither of these groups received the mailed outreach intervention but could have received FIT screening via usual care, which was at the discretion of the clinical care team in each clinic. FIT order placement by the patient’s PCP was captured during a 1-year look-forward period from the time of eligibility for CRC screening. FIT completion was also captured during the screening eligibility period. Colonoscopy was not included in the screening outcome because colonoscopy was not commonly utilized for screening in the health system; moreover, colonoscopy could be ordered for a symptomatic condition (ie anemia, weight loss). Patient vital status at 8-years of follow-up was assessed by examining the mortality data available in the electronic medical records.

### Analytic Plan

Patients who were not up to date with CRC screening were categorized as either having none of or at least 1 of the criteria defining laboratory abnormality. Patients who did not have any laboratory values measured were assumed to have normal laboratory values. Patient demographic characteristics were summarized using proportions or means and SDs and compared using Chi-square or t-tests, as appropriate.

The primary outcome compared the rate of FIT test ordered in patients with none vs at least 1 laboratory abnormality. Secondary outcomes included (1) the rate of FIT completion and (2) mortality. For the rate of FIT completion, we compared patients with no laboratory abnormality vs at least 1 laboratory abnormality. Both the primary outcome and the rate of FIT completion were collected during the 1-year look forward period following screening eligibility. FIT order placements and FIT completion rates were further evaluated according to each laboratory abnormality subgroup. Mortality was compared between each group at the 8-year follow-up from the initial study period. We did not adjust for protected traits such as sex and race/ethnicity, though they be associated with comorbidities, should not be used to triage screening invitations. All tests were 2-sided, and a *P* value of less than .05 was considered statistically significant.

## Results

### Patient Demographics

A total of 9676 patients were included in the analysis, of which 1053 met the criteria for at least 1 laboratory abnormality. The group flagged with any laboratory abnormality was more likely to be male compared to the group assigned to routine screening (71.2% vs 45.9%, *P* < .001) (Table 1). Race/ethnicity breakdown also differed between the 2 groups. The group without any laboratory abnormality was more likely Asian and preferred non-English language.

**Table 1:** Characteristics of the Study Populations.

Characteristic	Absence of Laboratory Abnormalities (*N* = 8623)	Presence of Laboratory Abnormalities* (*N* = 1053)
Sex—no. (%)		
Female	4661 (54.1)	303 (28.8)
Male	3962 (45.9)	750 (71.2)
Race/Ethnicity—no. (%)		
Hispanic	1774 (20.6)	218 (20.7)
Asian	3134 (36.3)	144 (13.7)
Non-Hispanic Black	1389 (16.1)	313 (29.7)
Non-Hispanic White	1604 (18.6)	276 (26.2)
Other	722 (8.4)	102 (9.7)
Preferred language—no. (%)		
Spanish	1263 (14.6)	90 (8.5)
English	4501 (52.2)	787 (74.7)
Chinese	1936 (22.5)	54 (5.1)
Other	923 (10.7)	122 (11.6)

⁎ Lab-based abnormalities including: albumin < 3 g/dL, HIV viral load > 10,000 copies or CD4 < 200 cells/µL, creatinine > 4 mg/dL, platelets < 100,000/µL, total bilirubin > 4 µmol/L, NH3 > 20, positive urine amphetamine or cocaine, serum ethanol > 80, haemoglobin A1C > 10%, and stage 3 or 4 cancer.

### FIT Order Placement Rates

**Figure 1:**
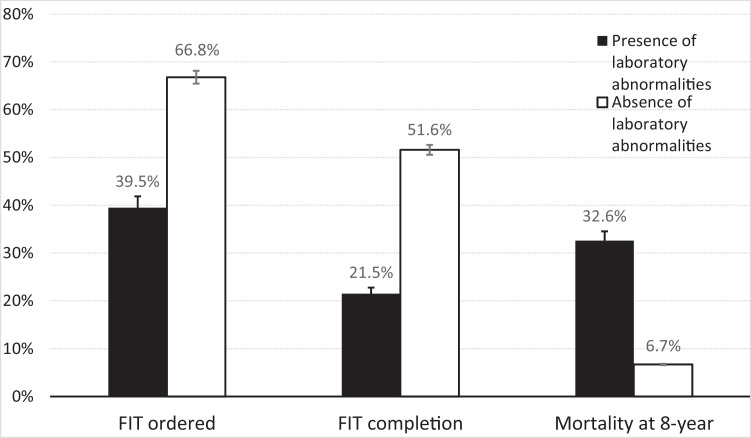
Overall frequency of FIT order, FIT completion, and mortality in patients with and without laboratory abnormalities.

The rate of FIT test ordered was significantly lower in patients who had at least 1 laboratory abnormality vs patients without any laboratory abnormalities (39.5% vs 66.8%, Odds Ratio [OR] 0.32, 95% CI, 0.28-0.37 *P* < .001) (Figure 1 and Table 2). Of the laboratory abnormalities, each subgroup had a significantly lower FIT order placement rate compared to the group without any laboratory abnormalities. The 1-year look forward order placement rates were significantly lower according to the following conditions: decompensated liver disease (platelets < 100,000/µL [36.8%], albumin < 3 g/dL [34.5%], total bilirubin > 4 µmol/L [39.2%], NH_3_ > 20 [37.7%]), AIDS or untreated HIV infection (HIV viral load > 10,000 copies [40.9%], CD4 < 200 cells/µL [35.7%]), advanced kidney disease (creatinine > 4 mg/dL [38%]), substance use disorder (positive urine amphetamine [41%], urine cocaine [38.9%]), uncontrolled diabetes (hemoglobin A1C > 10% [37.5%]), and regional or distant cancer (stage 3 cancer [27.3%], stage 4 cancer [36.8%]).

**Table 2:** FIT Order Status, FIT Completion, and Mortality Among All Patients with and Without Laboratory Abnormalities.

Characteristic	Participants, no.	FIT Ordered, no. (%)	Odds Ratio (95% CI)	FIT Completed, no. (%)	Odds Ratio (95% CI)	Mortality at 8-y, no. (%)	Odds Ratio (95% CI)
Absence of laboratory abnormalities	8623	5762 (66.8)	Reference	4448 (51.6)	Reference	580 (6.7)	Reference
Presence of laboratory abnormality	1053	416 (39.5)**	0.32 (0.28-0.37)	226 (21.5)**	0.26 (0.22-0.30)	343 (32.6)**	6.70 (5.74-7.81)
Albumin < 3 g/dL	255	88 (34.5)**	0.26 (0.20-0.34)	56 (21.9)**	0.26 (0.20-0.36)	75 (29.4%)**	5.78 (4.36-7.66)
CD4 < 200 cells/µL	140	50 (35.7)**	0.28 (0.19-0.39)	22 (15.7)**	0.18 (0.11-0.28)	47 (33.6)**	7.01 (4.86-10.05)
Creatinine > 4 mg/dL	179	68 (38.0)**	0.30 (0.22-0.41)	31 (17.3)**	0.20 (0.13-0.29)	79 (44.1)**	10.96 (8.06-14.89)
Platelets < 100,000/µL	274	101 (36.8)**	0.29 (0.23-0.37)	65 (23.7)**	0.29 (0.22-0.39)	90 (32.8)**	6.78 (5.20-8.85)
Total bilirubin > 4 µmol/L	125	49 (39.2)**	0.32 (0.22-0.46)	39 (31.2)**	0.43 (0.29-0.62)	28 (22.4)**	4.00 (2.61-6.15)
NH3 > 20	122	46 (37.7)**	0.30 (0.21-0.43)	30 (24.6) **	0.53 (0.29-0.97)	42 (34.4)**	7.28 (4.96-10.68)
Urine amphetamine	78	32 (41.0)**	0.35 (0.22-0.54)	13 (16.7)**	0.19 (0.10-0.34)	32 (41.0)**	9.65 (6.10-15.26)
Urine cocaine	95	37 (38.9)**	0.32 (0.21-0.48)	13 (13.7)**	0.15 (0.08-0.27)	38 (40.0)**	9.24 (6.08-14.06)
Serum ethanol > 80	10	3 (30.0)*	0.21 (0.05-0.82)	3 (30)	0.40 (0.10-1.56)	3 (30.0)**	5.94 (1.53-23.04)
Hgb A1c > 10%	24	9 (37.5)**	0.30 (0.13-0.68)	5 (20.8)**	0.25 (0.09-0.66)	10 (41.7)**	9.91 (4.38-22.40)
HIV viral load > 10,000 copies	66	27 (40.9)**	0.34 (0.21-0.56)	11 (16.7)**	0.19 (0.10-0.36)	25 (37.9)**	8.46 (5.11-14.00)
Stage 3 cancer	11	3 (27.3)**	0.18 (0.05-0.70)	1 (9.1)**	0.09 (0.01-0.73)	3 (27.3)**	5.20 (1.38-19.65)
Stage 4 cancer	19	7 (36.8)**	0.29 (0.11-0.74)	4 (21.1)**	0.25 (0.08-0.75)	7 (36.8)**	8.09 (3.17-20.52)

*P*-values: * *P* < .05, **P < .01.

### FIT Completion Rates

Similarly, FIT completion was significantly lower in patients who had at least 1 laboratory abnormality vs patients without any laboratory abnormalities (21.5% vs 51.6%, *P* < .001) (Figure 1 and Table 2). This corresponded to a 74% reduced odds of FIT test completion (OR 0.26, 95% CI, 0.22-0.30, *P* < .001). Each laboratory abnormality subgroup had a significantly lower rate of FIT completion compared to the group without any laboratory abnormality, similar in pattern to the FIT order placement rates: decompensated liver disease (platelets < 100,000/µL [23.7%], albumin < 3 g/dL [21.9%], total bilirubin > 4 µmol/L [31.2%], NH_3_ > 20 [24.6%]), AIDS or untreated HIV infection (HIV viral load > 10,000 copies [16.7%], CD4 < 200 cells/µL [15.7%]), advanced kidney disease (creatinine > 4 mg/dL [17.3%]), substance use disorder (positive urine amphetamine (16.7%), urine cocaine (13.7%)), uncontrolled diabetes (hemoglobin A1C > 10% [20.8%]), and regional or distant cancer (stage 3 cancer [9.1%], stage 4 cancer [21%]).

The FIT completion rate among those patients who had received a FIT order differed between the patient groups. Even when the FIT was ordered, patients with at least 1 laboratory abnormality were less likely to complete FIT testing compared to patients without any laboratory abnormalities (54.3% vs 77.2%, *P* < .001).

### Vital Status

Mortality at 8-year follow-up was significantly higher in patients who met at least 1 laboratory abnormality threshold vs patients without any laboratory abnormalities (32.6% vs 6.7%, OR 6.70, 95% CI, 5.74-7.81, *P* < .001) (Figure 1). Mortality status at 8-years was consistently elevated in these patients: decompensated liver disease (platelets < 100,000/µL [32.8%], albumin < 3 g/dL [32.6%], total bilirubin > 4 µmol/L [22.4%], NH_3_ > 20 [34.4%]), AIDS or untreated HIV infection (HIV viral load > 10,000 copies [37.9%], CD4 < 200 cells/µL [29.4%]), advanced kidney disease (creatinine > 4 mg/dL [44.1%]), substance use disorder (positive urine amphetamine [41%], urine cocaine [40%]), uncontrolled diabetes (hemoglobin A1C > 10% [41.7%]), and regional or distant cancer (stage 3 cancer [27.3%], stage 4 cancer [36.8%]).

## Discussion

In this cohort study of patients in a safety-net health system, we investigated the relationship between patients with and without a specified laboratory abnormality, as a surrogate for comorbid conditions, and their association with FIT orders, FIT completion rates, and mortality. Patients meeting an abnormal laboratory threshold experienced higher mortality and were both less likely to have FIT orders placed and less likely to complete FIT, suggesting that providers were less likely to offer screening, and patients were less likely to complete it. Among those with FIT ordered, completion rates significantly differed, which may reflect patient self-assessment of the risks and benefits of screening or other complex issues related to having comorbidities. Incorporating patient-level data could improve the delivery of an organized screening program and reduce overscreening.

Health systems, when reporting CRC screening measures, exclude patients from the measure if they have a history of total colectomy, history of colorectal cancer, history of inflammatory bowel disease, advanced illness diagnosis or frailty, dementia medications, or hospice. Beyond reporting, health system services should incorporate these factors in CRC outreach services to reduce overscreening. There is likely room for further improvement. Our study demonstrates that patient-level health data, including lab-based abnormalities, are a strong inverse predictor of CRC screening orders and completion, which differs from prior studies.^8^^,^^11^ In prior studies, health encounters were more frequent in patients with comorbid conditions, which appeared to have led to increased CRC screening completion; however, in this study, we observed significantly lower screening in our patients with laboratory abnormalities. While more visits with providers can increase screening, this may also be because colonoscopy may be coincidental with anemia and comorbidities, artificially increasing screening when completed, whereas FIT is primarily used for CRC screening and is less prone to use as a diagnostic study.

Prior studies also used the Charles-Deyo comorbidity score, which is an aggregate of comorbid conditions, whereas our study used lab values and the cancer registry, which is more likely to reflect active conditions. Prognosis calculators exist to guide shared decision-making regarding the benefits of screening and are available to clinicians in the outpatient setting to reduce overscreening.^12^^,^^13^ Population-based screening programs, which leverage structured health data to inform screening invitations in the absence of a provider-patient encounter, could benefit from similar ‘decision-making’ to refine screening invitations. Indeed, incorporating patient health data to inform CRC screening invitations could align population-based workflow and algorithms with the intent of primary care clinicians to encourage screening in those with perceived benefits of screening and reduce overscreening in those with little benefit or perceived risk.^14^ In the instance when a screening program defers a screening invitation, screening can still be initiated between the provider and patient.

There are several limitations of the current study. First, the patient population was derived from a safety-net population, largely comprised of Medicaid, low-income, undocumented, and uninsured patients. The prevalence of these factors is likely elevated compared with other settings, but we expect the association between reduced FIT completion and comorbidity to be similar across populations. Second, FIT could have been completed for indications other than CRC screening. For example, patients with significant comorbidities are more likely to be anemic or present to the emergency room, which could lead to FIT orders and completion. Thus, the true percentage of FIT orders and completion performed for screening is likely lower in patients with comorbid conditions. Third, the lab criteria evaluated for significant comorbidities in this study were not intended to be exhaustive; instead, the use of laboratory data and other criteria should be refined and further studied. Patients with serious cardiovascular or pulmonary comorbidity, or with extreme frailty, for example, might not have been captured by the criteria in this study. Fourth, we are unable to fully capture why screening did not take place though we acknowledge that health and lifestyle related behaviors are known to influence screening behavior. Although FIT order placement rates provide a useful piece of information in assessing provider intent to screen, chart reviews and qualitative interviews with patients and providers would be informative in understanding patient and provider intent to screen. Given the potential for incorrect attribution due to selection bias, data validity, and reliability, these results should be interpreted cautiously in the absence of provider input.

In summary, patient-level data can be used to identify subgroups of patients who harbor lab-based abnormalities associated with significant comorbidities and who are much less likely to complete CRC screening. Although causal inferences between laboratory abnormalities, screening receipt, and mortality cannot be drawn, given that missing data and residual confounding may influence the observed associations, future work will need to involve the PCP perspective in validating observed associations. Health systems and services that incorporate patient-level data can improve the delivery of an organized screening program, consistent with the intent of providers. Greater adoption and implementation of organized screening programs could improve overall screening rates.

## CRediT authorship contribution statement

**Rachel Corren:** Investigation, Formal analysis, Data curation. **Sylvia La:** Writing – review & editing, Resources, Investigation. **Edgar Corona:** Writing – review & editing, Methodology, Investigation. **Dalia Martinez:** Writing – review & editing, Software, Resources, Data curation. **Urmimala Sarkar:** Writing – review & editing, Resources. **Blake Gregory:** Writing – review & editing, Resources, Investigation. **Uri Ladabaum:** Writing – review & editing, Supervision, Investigation, Conceptualization. **Ma Somsouk:** Writing – review & editing, Writing – original draft, Visualization, Validation, Supervision, Investigation, Data curation, Conceptualization.

## Declaration of competing interest

The authors declare that they have no known competing financial interests or personal relationships that could have appeared to influence the work reported in this paper.

## References

[1] Colorectal Cancer: Screening. Recommendation: colorectal cancer: screening| United States preventive services taskforce, US Preventive Services Taskforce, Accessed May, 18 2021, Available at: www.uspreventiveservicestaskforce.org/uspstf/recommendation/colorectal-cancer-screening.

[2] Lee J.K., Liles E.G., Bent S., Levin T.R., Corley D.A. (2014 4). Accuracy of fecal immunochemical tests for colorectal cancer: systematic review and meta-analysis. Ann Intern Med.

[3] Somsouk M., Rachocki C., Mannalithara A. (2020). Effectiveness and cost of organized outreach for colorectal cancer screening: a randomized, controlled trial. J Natl Cancer Inst.

[4] Inadomi J.M., Vijan S., Janz N.K. (2012). Adherence to colorectal cancer screening: a randomized clinical trial of competing strategies. Arch Intern Med.

[5] Levin T.R., Corley D.A., Jensen C.D. (2018). Effects of organized colorectal cancer screening on cancer incidence and mortality in a large community-based population. Gastroenterology.

[6] Walter L.C., Covinsky K.E. (2001). Cancer screening in elderly patients: a framework for individualized decision making. JAMA.

[7] Calderwood A.H., Tosteson T.D., Wang Q., Onega T., Walter L.C. (2023). Association of life expectancy with surveillance colonoscopy findings and follow-up recommendations in older adults. JAMA Intern Med.

[8] Deardorff W.J., Lu K., Jing B. (2023). Frequency of screening for colorectal cancer by predicted life expectancy among adults 76-85 years. JAMA.

[9] (2022).

[10] Wang A., Lee B., Patel S., Whitaker E., Issaka R.B., Somsouk M. (2021). Selection of patients for large mailed fecal immunochemical test colorectal cancer screening outreach programs: a systematic review. J Med Screen.

[11] Walter L.C., Lindquist K., Nugent S. (2009). Impact of age and comorbidity on colorectal cancer screening among older veterans. Ann Intern Med.

[12] Lee S.J., Lindquist K., Segal M.R., Covinsky K.E. (2006). Development and validation of a prognostic index for 4-year mortality in older adults. JAMA.

[13] Yourman L.C., Lee S.J., Schonberg M.A., Widera E.W., Smith A.K. (2012). Prognostic indices for older adults: a systematic review. JAMA.

[14] Somsouk M., Lee B., Potter M. (2023). Opportunity and promise of stool-based organized colorectal cancer screening programs. Techniques Innovations Gastrointestinal Endosc.

